# Effects of antibacterial peptide-producing *Bacillus subtilis*, gallic acid, and cellulase on fermentation quality and bacterial community of whole-plant corn silage

**DOI:** 10.3389/fmicb.2022.1028001

**Published:** 2022-10-17

**Authors:** Zhiheng Zhang, Yuqin Wang, Saiqiao Wang, Lu Zhao, Binglei Zhang, Wanhang Jia, Zhenhan Zhai, Lingping Zhao, Yuanxiao Li

**Affiliations:** College of Animal Science and Technology, Henan University of Science and Technology, Luoyang, China

**Keywords:** whole-plant corn silage, *Bacillus subtilis*, gallic acid, cellulases, fermentation quality, bacterial community

## Abstract

In the current study, we assessed the effects of antibacterial peptide-producing *Bacillus subtilis* (BS), gallic acid (GA) and cellulase (CL) on the fermentation quality and bacterial community of various varieties of whole-plant corn silage. Three different varieties of whole-plant corn (Yuqing386, Enxiai298, and Nonghe35) were treated with 0.02% BS (fresh material basis), 0.2% GA (fresh material basis) and 0.02% CL (fresh material basis), after which 45 days of anaerobic fermentation were conducted. With the exception of its low dry matter content, the results showed that Yuqing386’s crude protein, water-soluble carbohydrate, and lactic acid contents were significantly higher than those of the other two corn varieties. However, its acid detergent fiber and cellulose contents were significantly lower than those of the other two corn varieties. Among the three corn variety silages, Yuqing386 had the highest relative abundance of *Lactobacillus* at the genus level and the biggest relative abundance of *Firmicutes* at the phylum level. In addition, the three additives markedly enhanced the quantity of dry matter and crude protein as compared to the control group. The application of GA considerably decreased the level of neutral detergent fiber while significantly increasing the content of lactic acid and water-soluble carbohydrates. Even though all additives enhanced the structure of the bacterial community following silage, the GA group experienced the greatest enhancement. On a phylum and genus level, the GA group contains the highest relative abundance of *Firmicutes* and *Lactobacillus*, respectively. Overall, of the three corn varieties, Yuqing386 provides the best silage qualities. GA has the biggest impact among the additions employed in this experiment to enhance the nutritional preservation and fermentation quality of whole-plant corn silage.

## Introduction

The moist forages can be effectively preserved using silage. Silage is produced by the fermentation of lactic acid bacteria (LAB) in anaerobic conditions, which can increase feed palatability, extend storage period, and reduce nutrient loss (Ni et al., 2017). Lactic acid bacteria have the ability to transform water-soluble carbohydrates (WSC) into organic acids, primarily lactic acid, during the ensiling process, lowering the pH, and preventing the growth of harmful microorganisms (Li and Nishino, 2011). Silage has always been a significant source of roughage for ruminant diets.

The manufacture of silage has made extensive use of crop feeds, including whole-plant corn, alfalfa, natural forages and others (Dunière et al., 2013). Additionally, whole-plant corn (WPC), which has a high biological yield and a bounty of water-soluble carbohydrates, has grown to be the most extensively utilized silage material globally (McDonald et al., 1991). WPC silage quality is influenced by a range of factors, including as variety, harvest stage, additives, and so on (Nazli et al., 2018; Wang et al., 2019a). Variety is a key component among them. According to Ferraretto et al. (2015) research, feeding dairy cows various types of corn silage had an impact on their consumption of dry matter, milk production, and starch digestibility. Varying types of WPC silage have different nutritional values and economic advantages, according to research by Nazli et al. (2018). To manage the quality of WPC silage, it is crucial to choose high-quality corn varieties.

The competition between undesirable microorganisms and lactic acid bacteria during the ensiling process, which may be significantly increased by employing additives, is what determines the quality of WPC silage (Wang et al., 2019a). Numerous silage additives have been researched and used in the production of silage for many years in order to further enhance the nutritional value and fermentation quality of silage (Muck et al., 2018). Based on their biochemical characteristics, silage additives can be classified as inoculants, chemicals, or enzymes (Sun et al., 2010). These additives are crucial for enhancing the microbial community of silages, nutritional value, and fermentation properties. Previous investigations have demonstrated that some metabolites of *Bacillus subtilis* (BS) can have antibacterial properties, and it can produce lactic acid by reducing pyruvate in anaerobic environment (Gandra et al., 2017; Lanna-Filho et al., 2017). In addition, BS and its metabolites can significantly increase the amount of lactic acid bacteria and aerobic stability in corn silages when used as a silage additive (Bonaldi et al., 2021). The natural organic acid gallic acid (GA), also known as 3,4,5-trihydroxybenzoic acid, has three phenolic hydroxyl groups and one carboxyl group (Badhani et al., 2015). According to reports, GA has broad-spectrum antibacterial activities, which might means that it can disintegrate the structural integrity of bacteria or prevent the development of bacterial biofilms (Kang et al., 2008; Díaz-Gómez et al., 2013). Additionally, GA can prevent protein hydrolysis during the ensiling process, which is an advantage of its polyhydroxy structure, which enhances protein binding (Jayanegara et al., 2019). He et al. (2020) reported that adding gallic acid to high- moisture mulberry leaves and stylo silage can improved the fermentation quality and protein preservation. Wang et al. (2021) also found that adding gallic acid to whole plant soybean silage was an effective strategy to protect feed nutrition and improve silage quality. As a consequence, GA could be the potential silage additive. Cellulase (CL) are currently the most popular enzyme in silage. In silage, CL primarily breaks down plant cell walls, releases a significant quantity of soluble sugar, and supplies a enough substrate for lactic acid bacteria fermentation (Muck et al., 2018). The combined addition of CL and galactosidase to alfalfa silage has been shown to dramatically lower the amount of ammonia nitrogen while increasing the amount of lactic acid and the relative abundance of lactic acid bacteria (Hu et al., 2021).

Few studies, to our knowledge, have looked into the impact of antibacterial peptide-producing BS and GA, on WPC silage. As a result, three varieties of corn, Yuqing386 (YQ), Enxiai298 (EXA), and Nonghe35 (NH) were ensiled in the current study with three different types of silage additives (BS, GA, and CL), and their chemical compositions, fermentation traits, and bacterial communities were evaluated after 45 days of fermentation. The purpose was to evaluate the application effect of antibacterial peptide-producing BS and GA in WPC silage, at the same time, high-quality corn varieties were selected. We intend to offer a theoretical foundation and statistical backing for the actual use of WPC silage.

## Materials and methods

### Raw materials and silage preparation

At the experimental teaching base of the College of Animal Science and Technology, Henan University of Science and Technology (Monsoon climate of medium latitudes: 34°35′N, 112°24′E, elevation 140 m, annual mean temperature 12.2–24.6°C, and average annual precipitation 528–800 mm), three varieties of corn, YQ, EXA, and NH, were planted on June 28, 2020 and harvested on October 7, 2020. Within an hour, a forage cutter (zengguang9zp-0.4, Zengguang group Co. Ltd., Yongkang, China) chopped the collected WPC into pieces measuring 1–2 cm. Following that, the chopped WPC were immediately ensiled with: (1) No additive (CK); (2) 0.02% *Bacillus subtilis* CP7 (BS) of fresh material (FM; Zhangye Aolin Beier Biological Technology Co, Ltd., China); (3) 0.2% Gallic acid (GA) of FM (Shanghai Yuanye Biological Technology Co. Ltd., China); and (4) 0.02% Cellulase (CL) of FM (Shanghai Yuanye Biological Technology Co. Ltd., China). After fully mixed, put the sample into a unidirectional exhaust fermentation bag (23 cm × 30 cm; Chenguang Shiye Co. Ltd., Wenzhou, China) in the amount of 500 g per bag. A total of 36 bags (3 varieties of raw material × 4 treatments × 3 replicates) were made and stored at room temperature and avoid light. After 45 days of ensiling, the chemical composition, fermentation characteristics, and bacterial community diversity were assessed.

### Chemical composition and fermentation characteristics analysis

After 45 days of ensiling, the silages and fresh materials were dried in a 65°C oven for 48 h, and then ground into powder with a knife mill (Zhejiang Yili Industry and Trade Co. Ltd., Zhejiang, China) through a 1 mm screen for subsequent layer analysis. The dry matter (DM) content of silages was measured by drying at 105°C in an electric blast drying oven for 4 h. The total nitrogen (TN) content was determined by Kjeldahl method (Krishnamoorthy et al., 1982; K1301, Chensheng Instrument Co. Ltd., Shanghai, China), and then the crude protein (CP) was equal to the TN × 6.25. Ether extract (EE), acid detergent lignin (ADL) and Ash shall be detected according to the procedures described by (AOAC, 2000). The contents of neutral detergent fiber (NDF) and acid detergent fiber (ADF) were determined with the ANKOM 2000i fiber analyzer (ANKOM 2000i, Anborui Science and Technology Co. Ltd., Beijing, China) according to the method of Van Soest et al. (1991). The contents of water-soluble carbohydrates (WSC) was determined by anthrone method (Murphy, 1958). The hemicellulose (HC) content was calculated by subtracting ADF from NDF, cellulose (CEL) content was the difference between ADF and ADL, and the holocellulose (HoC) content was the sum of HC and CEL (Ren et al., 2015). In addition, the biological degradation potential (BDP) was calculated by reference to Agneessens et al. (2015) using HoC divided by ADL.

Meanwhile, to determine the fermentation characteristics of silage, 20 g of each sample was weighed, added 180 ml distilled water, shake well, and then placed it in a refrigerator at 4°C for 24 h. After extraction, the filtrate was filtered through 4 layers of cheese cloth followed by 2 layers of filter paper. One aliquot of the filtrate was used to determine the pH of the filtrate immediately with a glass electrode pH meter (FE28, METTLER TOLEDO, Shanghai, China), and another aliquot was frozen at −20°C for the determination of organic acids and ammonia nitrogen (NH_3_-H). The content of NH_3_-N was determined by phenol hypochlorite colorimetry (Broderick and Kang, 1980). The content of lactic acid (LA) was determined by p-hydroxybiphenyl colorimetry method as the process described by Barnett (1951). Prior to examining the butyric acid (BA) and acetic acid (AA) concentrations, the filtrate was centrifuged at 10,000 rpm/min for 10 min, and the supernatant was detected by gas chromatography (GC-6800, Beifen Tianpu, Beijing, China). The analytical column was a quartz glass filled column (Φ 6 mm × 2 m), with a column temperature of 150°C and inlet temperature of 220°C; the injection volume was 1 μl; the FID detector temperature was 280°C; the carrier gas was high-purity N_2_ with a flow rate of 30 ml/min and a pressure of 200 kPa; the gas was H_2_ with a flow rate of 30 ml/min; the auxiliary gas was air with a flow rate of 300 ml/min. Finally, the flieg score (FS) was computed using equation given by Wang et al. (2017).

### 16S rDNA sequencing analysis

#### DNA extraction and PCR amplification

By using Power Soil DNA Isolation Kit (MO BIO Laboratories), total bacterial DNA was isolated from samples in line with the guidelines provided by the manufacturer. The ratios of 260 nm/280 nm and 260 nm/230 nm were used to evaluate the quality and amount of DNA. Following that, DNA was kept at −80°C until further processing. The common primer pair (Forward primer, 5′-ACTCCTACGGGAGGCAGCA-3′; Reverse primer, 5′-GGACTACHVGGGTWTCTAAT-3′) together with adapter sequences and barcode sequences were used to amplify the V3-V4 region of the bacterial 16S rRNA gene. 50 μl of a total volume were used for the PCR amplification, including 10 μM of each primer, 10 μl of buffer, 10 μl of high GC enhancer, 1 μl of dNTP, 0.2 μl of Q5 High-Fidelity DNA Polymerase, and 60 ng/μl of genomic DNA. Following a preliminary denaturation at 95°C for 5 min, there were 15 cycles of 95°C for 1 min, 50°C for 1 min, and 72°C for 1 min, with a final extension at 72°C for 7 min. Through the use of VAHTSTM DNA Clean Beads, the PCR products from the first step were purified. After then, a second round of PCR was carried out in a 40 μl reaction that comprised 10 μM of each primer, 20 μl of 2 × Phsion HF MM, 8 μl of ddH_2_O, and 10 μl PCR products from the previous round. Following a preliminary denaturation at 98°C for 30 s, there were 10 cycles of 98°C for 10 s, 65°C for 30 s, and 72°C for 30 s, with a final extension at 72°C for 5 min. Finally, Quant-iTTM dsDNA HS Reagent was used to quantify each PCR products and pool them all together. The purified, pooled sample was subjected to high-throughput sequencing analysis of bacterial rRNA genes on the Illumina Miseq 2500 platform (2 × 250 paired ends) at Beijing Tsingke Biotechnology Co, LTD in Beijing, China.

#### Bioinformatic analysis of sequencing data

Firstly, Trimmatic (version 0.33) was used to filter the quality of the raw reads, then FLASH (version 1.2.11) was used to splice double-ended reads and remove chimeras (UCHIME, version 8.1). Finally, high-quality sequences were obtained for subsequent analysis. Operational taxonomic units (OTUs) were clustered using USEARCH (version 10.0) with a 97% sequence similarity. By default, 0.005% of the number of sequences was used as the threshold to filter OTUs. The α-diversity was calculated by using QIIME2.^1^ R was also used to analyzed and display cluster heat maps, relative abundances of various microorganisms, and principal component analysis (PCA).

### Statistical analysis

The fermentation characteristics and chemical composition data of silages were analyzed by two-way ANOVA in SPSS software. The significant difference was then indicated at the level of *p* < 0.05 using Duncan’s multiple range tests to assess differences between treatments.

## Results

### Characteristics of fresh whole-plant corn of three different varieties before ensiling

The chemical compositions of the three different varieties of corn pre ensiling are shown in Table 1. The contents of several important indicators such as DM, CP, WSC, and NDF in YQ were 31.38% / FM, 8.02% / DM, 11.03% / DM, and 56.27% / DM, respectively. The contents of these components in EXA were DM (33.81% / FM), CP (8.57% / DM), WSC (9.86% / DM), and NDF (55.33% / DM). In NH, the contents of DM, CP, WSC, and NDF were 35.37% / FM, 7.85% / DM, 9.13% / DM, and 54.86% / DM, respectively.

**Table 1:** Chemical composition of fresh whole-plant corn of three different varieties before ensiling (±SD, *n* = 3).

Items	Corn varieties		
	YQ	EXA	NH
Dry matter / % FM	31.38 ± 3.37	33.81 ± 4.11	35.37 ± 3.94
Crude protein / % DM	8.02 ± 0.59	8.57 ± 0.30	7.85 ± 0.26
Ether extract / % DM	3.78 ± 0.66	3.82 ± 0.35	3.69 ± 0.28
Ash / % DM	4.90 ± 0.61	5.03 ± 0.76	4.60 ± 0.56
Acid detergent fiber / % DM	19.62 ± 1.46	21.39 ± 2.45	23.98 ± 2.04
Neutral detergent fiber / % DM	56.27 ± 2.08	55.33 ± 2.26	54.86 ± 3.59
Water soluble carbohydrate / % DM	11.03 ± 1.72	9.86 ± 1.32	9.13 ± 1.85

FM, fresh matter; DM, dry matter; YQ, corn variety Yuqing 386; EXA, corn variety Enxiai 298; NH, corn variety Nonghe35.

### Effects of varieties and additives on the chemical composition of whole-plant corn silage

The effects of varieties and additives on the chemical components of WPC silage such as DM, CP, EE, Ash, and WSC are presented in Table 2. When the variety was the main effect, EXA and NH had significantly larger DM contents than YQ (*p* < 0.05), the CP content in each treatment group showed that YQ and EXA were significantly higher than those of NH (*p* < 0.05). Only in CL treatment group, EE content showed that YQ was significantly less than EXA (*p* < 0.05). Ash content showed that there were significant differences among the three cultivars in GA treatment group (*p* < 0.05), and YQ > NH > EXA. WSC content in CK group YQ was significantly higher than EXA (*p* < 0.05), and there were significant differences in GA and CL groups (*p* < 0.05), and the expression was YQ > EXA > NH. When the additive was the main effect, in YQ and NH, the content of DM in each additive group was significantly higher than that in the CK group (*p* < 0.05). In YQ and EXA, the content of CP in each additive was significantly higher than those in the CK group (*p* < 0.05). In NH, the content of CP in GA and CL groups were significantly higher than BS and CK groups (*p* < 0.05); EE only in YQ, CL groups were significantly lower than BS and CK groups (*p* < 0.05); WSC in YQ, the GA and CL groups were significantly higher than the CK group (*p* < 0.05); and the BS group was significantly lower than the CK group (*p* < 0.05). In EXA and NH, the content of WSC in GA group was significantly higher than the CL group (*p* < 0.05), BS and CK groups were significantly lower than the CL group (*p* < 0.05). And the interaction effect of varieties and additives had a significant effect on CP of WPC silage (*p* < 0.05), had a highly significant effect on WSC (*p* < 0.01) but not significant effect on DM, EE and Ash (*p* > 0.05).

**Table 2:** Effects of varieties and additives on the chemical compositions of whole-plant corn silage (±SD, *n* = 3).

Items	Treatments	Varieties			Significance		
		YQ	EXA	NH	V	T	V × T
^2^Dry matter / % FM	CK	^1^27.59 ± 0.06^Aa^	31.93 ± 0.16^Ca^	30.84 ± 0.10^Ba^	**	**	NS
	BS	29.82 ± 0.24^Ab^	32.47 ± 0.44^Ba^	32.33 ± 0.26^Bab^			
	GA	30.31 ± 0.38^Ab^	33.58 ± 0.17^Ba^	33.87 ± 0.46^Bb^			
	CL	31.09 ± 0.30^Ab^	32.27 ± 0.29^ABa^	33.42 ± 0.19^Bb^			
Crude protein / % DM	CK	8.11 ± 0.18^Ba^	8.39 ± 0.10^Ba^	7.00 ± 0.14^Aa^	**	**	*
	BS	9.90 ± 0.20^Cb^	8.96 ± 0.02^Bb^	7.07 ± 0.14^Aa^			
	GA	9.81 ± 0.12^Bb^	9.53 ± 0.06^Bc^	8.77 ± 0.13^Ab^			
	CL	9.72 ± 0.24^Bb^	9.68 ± 0.13^Bc^	8.16 ± 0.09^Ab^			
	CK	3.85 ± 0.11^Ab^	3.39 ± 0.08^Aa^	3.72 ± 0.15^Aa^	NS	NS	NS
Ether extract / % DM	BS	3.72 ± 0.10^Ab^	3.29 ± 0.06^Aa^	3.40 ± 0.11^Aa^			
	GA	3.39 ± 0.07^Aab^	3.31 ± 0.05^Aa^	3.65 ± 0.17^Aa^			
	CL	3.04 ± 0.01^Aa^	3.55 ± 0.10^Ba^	3.40 ± 0.07^ABa^			
Ash / % DM	CK	5.16 ± 0.09^Aa^	6.29 ± 1.22^Aa^	4.52 ± 0.04^Aa^	NS	NS	NS
	BS	4.85 ± 0.09^Aa^	5.11 ± 0.27^Aa^	5.08 ± 0.06^Aa^			
	GA	5.33 ± 0.12^Ca^	3.57 ± 0.07^Aa^	4.52 ± 0.10^Ba^			
	CL	4.97 ± 0.16^Aa^	4.45 ± 0.17^Aa^	4.85 ± 0.10^Aa^			
Water soluble carbohydrate / % DM	CK	4.51 ± 0.08^Bb^	4.11 ± 0.24^Aa^	4.28 ± 0.16^ABa^	**	**	**
	BS	4.07 ± 0.23^Aa^	4.11 ± 0.18^Aa^	4.21 ± 0.10^Aa^			
	GA	6.48 ± 0.04^Cc^	6.04 ± 0.04^Bc^	5.18 ± 0.27^Ac^			
	CL	6.55 ± 0.03^Cc^	5.52 ± 0.17^Bb^	4.85 ± 0.08^Ab^			

1 Capital letters indicate significant differences between varieties under the same treatment (*p* < 0.05). Lowercase letters indicate significant differences among different treatments of the same variety (*p* < 0.05).

^2^FM, fresh matter; DM, dry matter; YQ, corn variety Yuqing 386; EXA, corn variety Enxiai 298; NH, corn variety Nonghe35; CK, no additives; BS, 0.02% *Bacillus subtilis* CP7 of FM; GA, 0.2% gallic acid of FM; CL, 0.02% cellulase of FM; V, corn varieties effect; T, treatments effect; V × T, the interaction effect of treatments and corn varieties. *p < 0.05; **p < 0.01; NS, no significant effect.

### Effects of varieties and additives on lignocellulosic composition of whole-plant corn silage

The effects of varieties and additives on the lignocellulosic components of WPC silage such as NDF, ADF, ADL, CEL, HC, and HoC are demonstrated in Table 3. When variety was the main effect, the content of NDF and HoC did not differ significantly between varieties (*p* > 0.05); the content of ADF and CL were not significantly different among the varieties in the CK group (*p* > 0.05), in the other treatment groups, YQ and EXA were significantly lower than NH (*p* < 0.05); The ADL content of YQ and EXA was significantly higher than that of NH only in the GA group (*p* < 0.05); the HC content of YQ and EXA was significantly higher than that of NH only in the BS group (*p* < 0.05). When the additive was the main effect, the NDF content in YQ only showed that the GA group was significantly lower than the other treatment groups (*p* < 0.05). The ADF content in YQ showed that the CL group was significantly lower than the BS and CK groups (*p* < 0.05), and in EXA, the CL and GA groups were significantly lower than the BS and CK groups (*p* < 0.05). ADL in YQ, the CL group was significantly lower than the BS group (*p* < 0.05), and in NH, the additives groups were significantly lower than the CK group (*p* < 0.05). Only in EXA, the CEL content of the GA and CL groups was significantly lower than that of the CK group (*p* < 0.05); the HoC content only in YQ, the GA and CL groups were significantly lower than the CK group (*p* < 0.05). HC content in different treatments groups showed no significant difference (*p* > 0.05). The interaction between varieties and additives only had a significant effect on CEL content (*p* < 0.05).

**Table 3:** Effects of varieties and additives on lignocellulosic compositions of whole-plant corn silage (±SD, *n* = 3).

Items	Treatments	Varieties			Significance		
		YQ	EXA	NH	V	T	V × T
^2^Neutral detergent fiber / % DM	CK	^1^51.93 ± 1.22^Ab^	50.07 ± 2.46^Aa^	49.40 ± 2.16^Aa^	NS	NS	NS
	BS	50.31 ± 0.33^Aab^	48.45 ± 1.33^Aa^	49.21 ± 0.47^Aa^			
	GA	45.47 ± 0.97^Aa^	47.84 ± 1.55^Aa^	49.37 ± 0.63^Aa^			
	CL	46.83 ± 0.72^Aab^	46.63 ± 1.47^Aa^	48.30 ± 0.20^Aa^			
Acid detergent fiber / % DM	CK	18.83 ± 0.52^Ab^	20.74 ± 1.09^Ab^	21.64 ± 0.65^Aa^	**	**	NS
	BS	18.50 ± 0.80^Ab^	18.01 ± 0.60^Aab^	22.09 ± 0.17^Ba^			
	GA	17.27 ± 0.38^Aab^	14.08 ± 0.64^Aa^	22.55 ± 0.84^Ba^			
	CL	15.28 ± 0.33^Aa^	14.42 ± 0.29^Aa^	20.16 ± 0.57^Ba^			
Acid detergent lignin / % DM	CK	2.99 ± 0.22^Aab^	3.16 ± 0.22^Aa^	3.68 ± 0.14^Ab^	*	**	NS
	BS	3.64 ± 0.02^Ab^	3.05 ± 0.27^Aa^	2.68 ± 0.14^Aa^			
	GA	3.34 ± 0.05^Bab^	2.70 ± 0.16^ABa^	2.31 ± 0.07^Aa^			
	CL	2.82 ± 0.10^Aa^	2.38 ± 0.16^Aa^	2.08 ± 0.14^Aa^			
Cellulose / % DM	CK	15.84 ± 0.57^Aa^	17.58 ± 0.89^Ab^	17.96 ± 0.56^Aa^	**	**	*
	BS	14.87 ± 0.80^Aa^	14.97 ± 0.44^Aab^	19.41 ± 0.18^Ba^			
	GA	13.93 ± 0.40^Aa^	11.38 ± 0.60^Aa^	20.25 ± 0.78^Ba^			
	CL	12.45 ± 0.41^Aa^	12.04 ± 0.41^Aa^	18.08 ± 0.69^Ba^			
Hemicellulose / % DM	CK	33.10 ± 1.48^Aa^	29.32 ± 3.29^Aa^	27.76 ± 2.29^Aa^	NS	NS	NS
	BS	31.81 ± 0.49^Ba^	30.43 ± 0.98^ABa^	27.12 ± 0.63^Aa^			
	GA	28.20 ± 0.70^Aa^	33.76 ± 1.38^Aa^	26.82 ± 1.46^Aa^			
	CL	31.56 ± 0.46^Aa^	32.21 ± 1.71^Aa^	28.14 ± 0.59^Aa^			
Holocellulose / % DM	CK	48.94 ± 1.02^Ab^	46.91 ± 2.67^Aa^	45.72 ± 2.27^Aa^	NS	NS	NS
	BS	46.67 ± 0.34^Aab^	45.4 ± 1.34^Aa^	46.53 ± 0.58^Aa^			
	GA	42.13 ± 0.96^Aa^	45.14 ± 1.39^Aa^	47.06 ± 0.70^Aa^			
	CL	44.01 ± 0.76^Aa^	44.25 ± 1.31^Aa^	46.22 ± 0.30^Aa^			

1 Capital letters indicate significant differences between varieties under the same treatment (*p* < 0.05). Lowercase letters indicate significant differences among different treatments of the same variety (*p* < 0.05).

^2^FM, fresh matter; DM, dry matter; YQ, corn variety Yuqing 386; EXA, corn variety Enxiai 298; NH, corn variety Nonghe35; CK, no additives; BS, 0.02% *Bacillus subtilis* CP7 of FM; GA, 0.2% gallic acid of FM; CL, 0.02% cellulase of FM; V, corn varieties effect; T, treatments effect; V × T, the interaction effect of treatments and corn varieties. *p < 0.05; **p < 0.01; NS, no significant effect.

### Effects of varieties and additives on fermentation quality of whole-plant corn silage

The effects of varieties and additives on the fermentation quality of WPC silage such as pH, LA, AA, PA, BA, and NH_3_-N / TN were displayed in Table 4. When variety was the main effect, the pH value only in the CL group showed that EXA and NH were significantly lower than YQ (*p* < 0.05); the LA content of the three corn varieties in the CK and CL groups showed significant difference (*p* < 0.05), and YQ > NH > EXA, in the BS group, YQ and NH were significantly higher than EXA (*p* < 0.05). There was no significant difference in AA content among different varieties in each treatment group (*p* > 0.05); BA content in CK group and GA group showed that YQ was significantly higher than the other two varieties (*p* < 0.05), and in CL group showed that YQ was significantly higher than NH (*p* < 0.05). NH_3_-N / TN in both GA and CK groups showed that YQ was significantly higher than EXA and NH (*p* < 0.05), and in BS group the performance of YQ was significantly higher than that of EXA (*p* < 0.05). When the additive was the main effect, the pH value of the GA group was significantly lower than that of the BS group only in EXA (*p* < 0.05). The LA content in YQ showed that the BS group was significantly higher than the GA and CL groups (*p* < 0.05), and the CK group was significantly lower than the GA and CL groups (*p* < 0.05), in EXA, all treatments showed significant differences (*p* < 0.05), and GA group > BS group > CL group > CK group, in NH, BS. and GA groups were significantly higher than CL and CK groups (*p* < 0.05). There was no significant difference in the contents of AA and BA among different treatment groups in each variety (*p* > 0.05); NH_3_-N / TN in YQ showed that CL group was significantly lower than BS and CK groups (*p* < 0.05), and GA group was significantly lower than the CK group (*p* < 0.05), in EXA, the GA group was significantly lower than the BS and CK groups (*p* < 0.05), and in NH, the GA, and CL groups were significantly lower than the BS and CK group (*p* < 0.05). The interaction of varieties and additives only had a very significant effect on LA content (*p* < 0.01). PA was not detected in all groups in this experiment.

**Table 4:** Effects of varieties and additives on fermentation quality of whole-plant corn silage (±SD, *n* = 3).

Items	Treatments	Varieties			Significance		
		YQ	EXA	NH	V	T	V × T
^2^pH	CK	^1^3.88 ± 0.01^Aa^	3.91 ± 0.02^Aab^	3.92 ± 0.01^Aa^	NS	NS	NS
	BS	3.90 ± 0.00^Aa^	3.96 ± 0.01^Ab^	3.91 ± 0.02^Aa^			
	GA	3.87 ± 0.02^Aa^	3.87 ± 0.01^Aa^	3.93 ± 0.01^Aa^			
	CL	3.90 ± 0.00^Aa^	3.93 ± 0.00^Bab^	3.93 ± 0.01^Ba^			
Lactic acid / (mg.g^−1^ DM)	CK	67.08 ± 0.28^Ca^	47.82 ± 0.41^Aa^	64.30 ± 0.46^Bb^	**	**	**
	BS	73.43 ± 0.63^Bc^	60.66 ± 0.08^Ac^	73.44 ± 0.43^Bc^			
	GA	69.65 ± 0.26^Ab^	71.8 ± 0.70^Ad^	72.35 ± 0.59^Ac^			
	CL	69.68 ± 0.41^Cb^	58.06 ± 0.41^Ab^	61.01 ± 0.50^Ba^			
Acetic acid / (mg.g^−1^ DM)	CK	7.49 ± 0.36^Aa^	6.08 ± 0.26^Aa^	7.43 ± 0.32^Aa^	NS	NS	NS
	BS	6.83 ± 0.57^Aa^	6.50 ± 0.14^Aa^	7.49 ± 0.39^Aa^			
	GA	5.95 ± 0.14^Aa^	6.14 ± 0.16^Aa^	7.08 ± 0.36^Aa^			
	CL	5.80 ± 0.33^Aa^	6.46 ± 0.56^Aa^	6.72 ± 0.11^Aa^			
Propionic acid / (mg.g^−1^ DM)	CK	ND	ND	ND	—	—	—
	BS	ND	ND	ND			
	GA	ND	ND	ND			
	CL	ND	ND	ND			
Butyric acid / (mg.g^−1^ DM)	CK	0.20 ± 0.01^Ba^	0.04 ± 0.01^Aa^	0.06 ± 0.01^Aa^	**	NS	NS
	BS	0.07 ± 0.02^Aa^	ND	0.05 ± 0.01^Aa^			
	GA	0.17 ± 0.01^Ba^	0.03 ± 0.00^Aa^	0.05 ± 0.01^Aa^			
	CL	0.17 ± 0.04^Ba^	ND	0.05 ± 0.00^Aa^			
NH_3_-N / TN / %	CK	5.03 ± 0.23^Bc^	3.33 ± 0.17^Abc^	3.82 ± 0.05^Ab^	**	NS	NS
	BS	4.75 ± 0.12^Bbc^	3.70 ± 0.15^Ac^	4.01 ± 0.19^ABb^			
	GA	3.66 ± 0.22^Bab^	2.31 ± 0.08^Aa^	2.59 ± 0.19^Aa^			
	CL	3.21 ± 0.28^Aa^	2.73 ± 0.20^Aab^	2.82 ± 0.11^Aa^			

1 Capital letters indicate significant differences between varieties under the same treatment (*p* < 0.05). Lowercase letters indicate significant differences among different treatments of the same variety (*p* < 0.05).

^2^FM, fresh matter; DM, dry matter; YQ, corn variety Yuqing 386; EXA, corn variety Enxiai 298; NH, corn variety Nonghe35; CK, no additives; BS, 0.02% *Bacillus subtilis* CP7 of FM; GA, 0.2% gallic acid of FM; CL, 0.02% cellulase of FM; V, corn varieties effect; T, treatments effect; V × T, the interaction effect of treatments and corn varieties. *p < 0.05; **p < 0.01; NS, no significant effect.

### Effects of varieties and additives on BDP and FS of whole-plant corn silage

**Figure 1:**
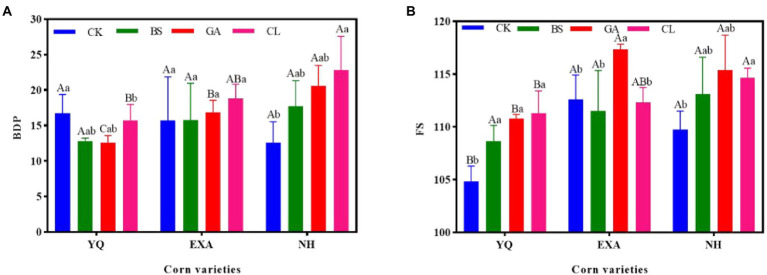
Effects of Varieties and Additives on **(A)** BDP and **(B)** FS of Whole-plant corn silage. BDP, biological degradation potential; FS, flieg score; YQ, corn variety Yuqing 386; EXA, corn variety Enxiai 298; NH, corn variety Nonghe35; CK, no additives; BS, 0.02% *Bacillus subtilis* CP7 of FM; GA, 0.2% gallic acid of FM; CL, 0.02% cellulase of FM; capital letters indicate significant differences between varieties under the same treatment (*p* < 0.05). Lowercase letters indicate significant differences among different treatments of the same variety (*p* < 0.05).

The effects of varieties and additives on BDP and FS of WPC silage are presented in Figure 1. When variety was the main effect, BDP showed significant differences among the three varieties in the GA group (*p* < 0.05), and NH > EXA > YQ, in the CL group, showed NH and EXA were significantly higher than YQ (*p* < 0.05). The FS of YQ in both CK and GA groups was significantly lower than that of EXA and NH (*p* < 0.05). When additive was the main effect, BDP in YQ, CL and CK groups were significantly higher than BS and GA groups (*p* < 0.05), and in NH, GA, and CL groups were significantly higher than CK groups (*p* < 0.05). FS in YQ, each additive group was significantly higher than CK group (*p* < 0.05); in EXA, GA group was significantly higher than the other groups (*p* < 0.05); in NH, GA, and CL group was significantly higher than the CK group (*p* < 0.05).

### Diversity of the bacterial community in whole-plant corn silage with different treatments

**Figure 2:**
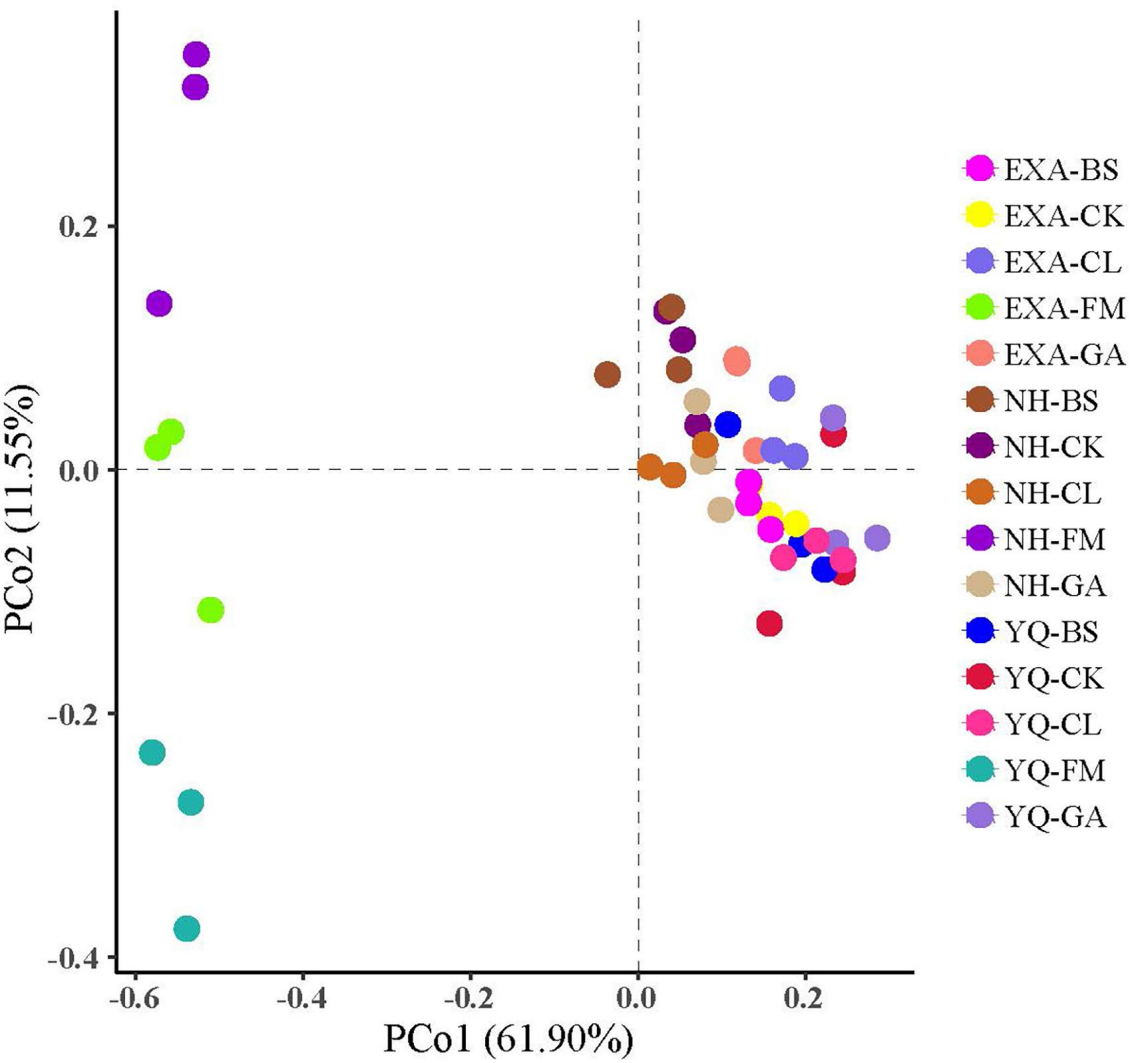
Principal component analysis (PCA) of bacterial communities for whole-plant corn silage with different treatmentsn. YQ, corn variety Yuqing 386; EXA, corn variety Enxiai 298; NH, corn variety Nonghe35; FM, fresh raw materials; CK, no additives; BS, 0.02% *Bacillus subtilis* CP7 of FM; GA, 0.2% gallic acid of FM; CL, 0.02% cellulase of FM.

The α-diversity of bacterial communities in fresh materials and WPC silage was displayed in Table 5 of this study. The reads in each group ranged from 77,928 to 48,656, with the highest reads in the YQ-CL group (77,928) and the lowest in the YQ-FM group (48,656), with an average of 67,172. Shannon and Simpson showed that it was significantly lower than that of fresh corn material after silage (*p* < 0.05); Shannon showed the highest NH-FM and the lowest YQ-GA in all groups; Simpson showed the highest EXA-FM and the lowest YQ-GA. Among the three corn varieties, OTUs, ACE, Chao1, Shannon, and Simpson of YQ were significantly lower than the other two varieties (*p* < 0.05). The interaction of varieties and additives had very significant effect on OTUs, ACE, Chao1 and Simpson (*p* < 0.01). The Coverage of each group in this experiment was above 99%, indicating that the sequencing results represented the real situation of the microorganisms in the samples. The results of β-diversity analysis of fresh raw materials and WPC silage are shown in Figure 2, it could be seen from the principal component analysis (PCA) that PCo 1 and PCo 2 were 61.90 and 11.55%, respectively. At the same time, the bacterial communities of the fresh raw materials of the three corn varieties were separated from each other, and the bacterial communities of the fresh raw materials and WPC silage were also significantly separated.

**Table 5:** Alpha diversity of bacterial communities in whole-plant corn silage with different treatments.

Items	Treatments	Varieties			Significance		
		YQ	EXA	NH	V	T	V × T
^2^OTUs	FM	^1^268.67 ± 4.04^Aab^	454.00 ± 4.64^Bb^	496.33 ± 1.37^Cc^	**	**	**
	CK	236.67 ± 20.27^Aa^	394.33 ± 2.31^Ba^	480.00 ± 1.70^Bbc^			
	BS	327.33 ± 5.72^Ab^	440.33 ± 14.03^Bab^	469.00 ± 8.18^Bb^			
	GA	322.33 ± 1.81^Ab^	468.00 ± 3.68^Cb^	416.33 ± 1.29^Ba^			
	CL	322.00 ± 6.69^Ab^	445.67 ± 2.78^Cb^	395.00 ± 1.70^Ba^			
ACE	FM	294.41 ± 4.68^Aa^	464.35 ± 3.77^Bb^	500.27 ± 1.30^Cb^	**	*	**
	CK	300.44 ± 21.05^Aa^	430.19 ± 2.48^Ba^	490.51 ± 2.09^Bb^			
	BS	383.49 ± 2.51^Ab^	467.46 ± 9.16^Bb^	480.98 ± 5.46^Bb^			
	GA	384.60 ± 9.41^Ab^	481.46 ± 1.39^Bb^	445.00 ± 1.78^Ba^			
	CL	380.45 ± 2.55^Ab^	459.76 ± 2.55^Cab^	428.99 ± 2.77^Ba^			
Chao1	FM	306.19 ± 7.82^Aab^	468.49 ± 2.89^Bab^	502.18 ± 1.22^Bb^	**	NS	**
	CK	300.08 ± 24.22^Aa^	438.87 ± 4.38^Ba^	496.65 ± 3.28^Bb^			
	BS	382.80 ± 3.05^Aab^	477.53 ± 6.78^Bb^	487.42 ± 5.55^Bb^			
	GA	392.30 ± 9.73^Ab^	484.96 ± 1.07^Bb^	447.86 ± 1.73^Ba^			
	CL	377.00 ± 3.23^Aab^	467.66 ± 3.92^Cab^	434.18 ± 4.22^Ba^			
Shannon	FM	4.33 ± 0.21^Ab^	6.15 ± 0.12^Bc^	6.30 ± 0.28^Bb^	**	**	NS
	CK	1.57 ± 0.09^Aa^	2.27 ± 0.06^Ba^	3.57 ± 0.08^Ca^			
	BS	2.06 ± 0.17^Aa^	2.51 ± 0.07^Bab^	4.05 ± 0.06^Ba^			
	GA	1.23 ± 0.08^Aa^	2.85 ± 0.07^Bb^	3.24 ± 0.05^Ba^			
	CL	1.62 ± 0.12^Aa^	2.46 ± 0.05^Bab^	3.49 ± 0.02^Ca^			
Simpson	FM	0.87 ± 0.02^Ac^	0.96 ± 0.00^Ac^	0.93 ± 0.02^Ac^	**	**	**
	CK	0.35 ± 0.03^Aab^	0.49 ± 0.01^Ba^	0.71 ± 0.01^Cab^			
	BS	0.48 ± 0.04^Ab^	0.53 ± 0.01^Aab^	0.75 ± 0.01^Bb^			
	GA	0.24 ± 0.02^Aa^	0.56 ± 0.01^Bb^	0.65 ± 0.00^Ca^			
	CL	0.34 ± 0.03^Aab^	0.53 ± 0.01^Bab^	0.74 ± 0.00^Cb^			
Coverage	FM	0.9990	0.9996	0.9998	*—*	*—*	*—*
	CK	0.9989	0.9992	0.9995			
	BS	0.9987	0.9993	0.9996			
	GA	0.9986	0.9995	0.9993			
	CL	0.9991	0.9994	0.9993			
Reads	FM	48,656	65,471	51,324	*—*	*—*	*—*
	CK	68,503	76,053	67,463			
	BS	62,553	73,266	67,362			
	GA	65,463	65,044	74,515			
	CL	77,928	69,011	74,961			

1 Capital letters indicate significant differences between varieties under the same treatment (*p* < 0.05). Lowercase letters indicate significant differences among different treatments of the same variety (*p* < 0.05).

^2^FM, fresh matter; DM, dry matter; YQ, corn variety Yuqing 386; EXA, corn variety Enxiai 298; NH, corn variety Nonghe35; CK, no additives; BS, 0.02% *Bacillus subtilis* CP7 of FM; GA, 0.2% gallic acid of FM; CL, 0.02% cellulase of FM; V, corn varieties effect; T, treatments effect; V × T, the interaction effect of treatments and corn varieties. *p < 0.05; **p < 0.01; NS, no significant effect.

### Bacterial relative abundance of whole-plant corn silage with different treatments

**Figure 3:**
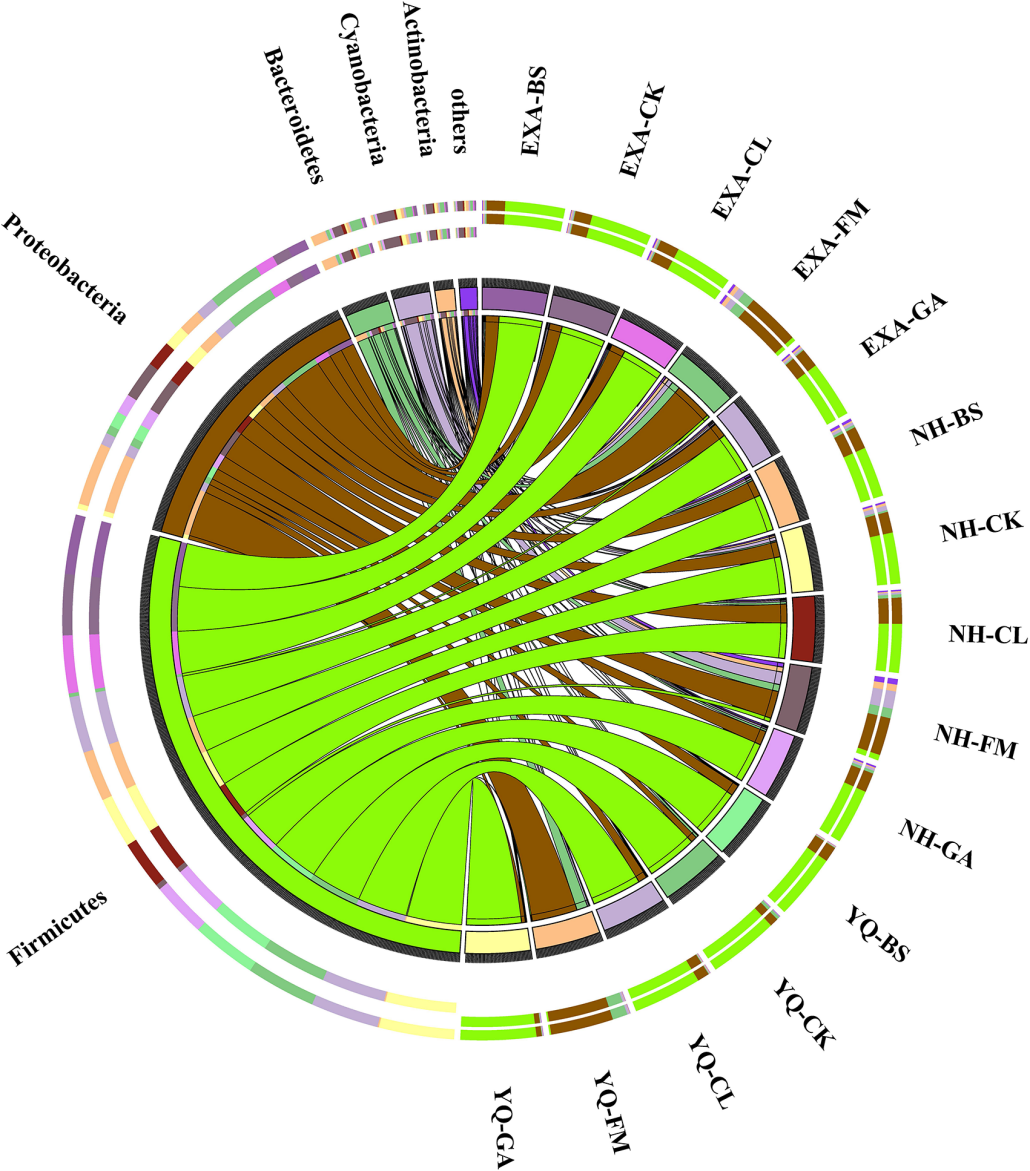
Circos map of bacterial communities at the phylum for whole-plant corn silage with different treatments. YQ, corn variety Yuqing 386; EXA, corn variety Enxiai 298; NH, corn variety Nonghe35; FM, fresh raw materials; CK, no additives; BS, 0.02% *Bacillus subtilis* CP7 of FM; GA, 0.2% gallic acid of FM; CL, 0.02% cellulase of FM.

The relative abundance of bacterial communities for fresh raw material and WPC silage with different treatments at the phylum is presented in Figure 3 (Circos map). As seen by the Circos map, *Proteobacteria*, *Bacteroidetes*, *Cyanobacteria*, *Firmicutes*, and *Actinobacteria* were the top five phyla bacteria attached to the 3 fresh corn raw materials. The relative abundances of the top five phyla that attached to YQ-FM were *Proteobacteria* (76.08%), *Bacteroidetes* (18.35%), *Cyanobacteri*a (2.68%), *Firmicutes* (1.73%) and *Actinobacteria* (0.66%), respectively; The relative abundances of the top five phyla that attached to EXA-FM were *Proteobacteria* (64.88%), *Bacteroidetes* (14.09%), *Cyanobacteria* (8.10%), *Actinobacteria* (5.27%), and *Firmicutes* (4.44%), respectively. The relative abundances of the top five phyla that attached to NH-FM were *Proteobacteria* (45.69%), *Cyanobacteria* (21.15%), *Bacteroidetes* (11.69%), *Actinobacteria* (8.29%), and *Firmicutes* (6.40%), respectively. After fermentation, the dominant phylum in each treatment group was *Firmicutes*. In YQ, the GA group had the highest relative abundance of *Firmicutes*, and the BS group had the lowest relative abundance of *Firmicutes*, at 91.47 and 79.10%, respectively. In EXA, the relative abundance of *Firmicutes* was the maximum in the CK group and the minimum in the GA group, 73.86 and 68.60%, respectively. In NH, the relative abundance of *Firmicutes* was the highest in the GA group and the lowest in the CL group, 66.80 and 59.02%, respectively. The relative abundance of *Firmicutes* in the three varieties WPC silage was YQ > EXA > NH. After fermentation, each treatment group’s relative abundance of *Proteobacteria* declined noticeably, and the relative abundance of *Bacteroidetes* likewise trended lower. In YQ, the BS group had the largest relative abundance of *proteobacteria*, that was 18.40%, while the GA group had the lowest relative abundance, which was 6.38%; In EXA, the relative abundance of *Proteobacteria* was the highest in the CL group and the lowest in the CK group, 23.71 and 20.74%, respectively; In NH, the relative abundance of *Proteobacteria* in the CL group was the highest, and the GA group was the lowest, 31.57 and 23.62%, respectively; And the relative abundance of *Proteobacteria* in the three varieties WPC silage was NH > EXA > YQ. The relative abundance of *Bacteroidetes* was reduced to less than 1% in YQ by the application of several additives; In EXA and NH, the effects of each additive were reversed, increasing the relative abundance of *Bacteroidetes* bacteria to more than 1%; while the relative abundance of *Bacteroidetes* bacteria in the three species of WPC silage was ranked NH > YQ > EXA.

**Figure 4:**
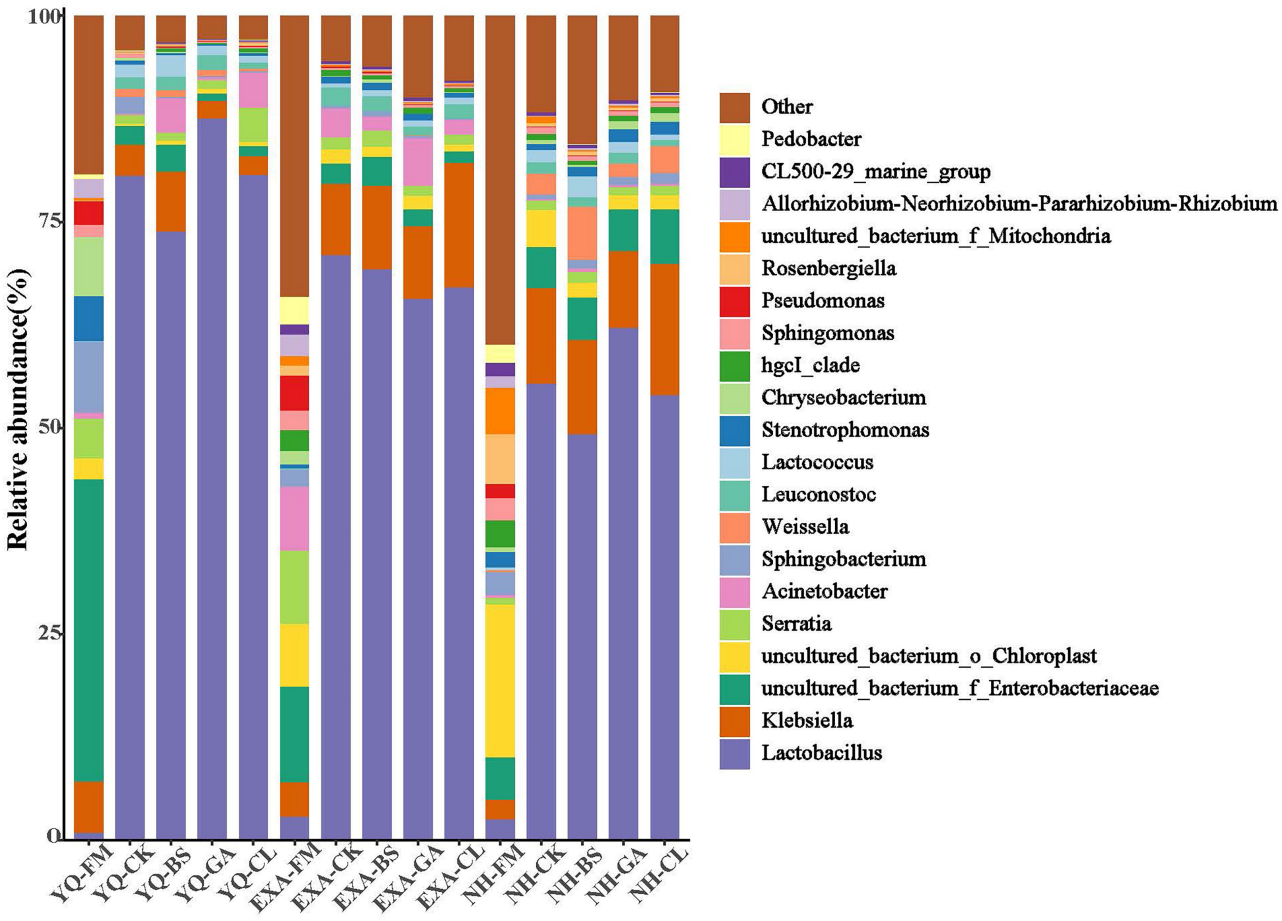
Relative abundance of bacterial communities at the genus levels for whole-plant corn silage with different treatments. YQ, corn variety Yuqing 386; EXA, corn variety Enxiai 298; NH, corn variety Nonghe35; FM, fresh raw materials; CK, no additives; BS, 0.02% *Bacillus subtilis* CP7 of FM; GA, 0.2% gallic acid of FM; CL, 0.02% cellulase of FM.

**Figure 5:**
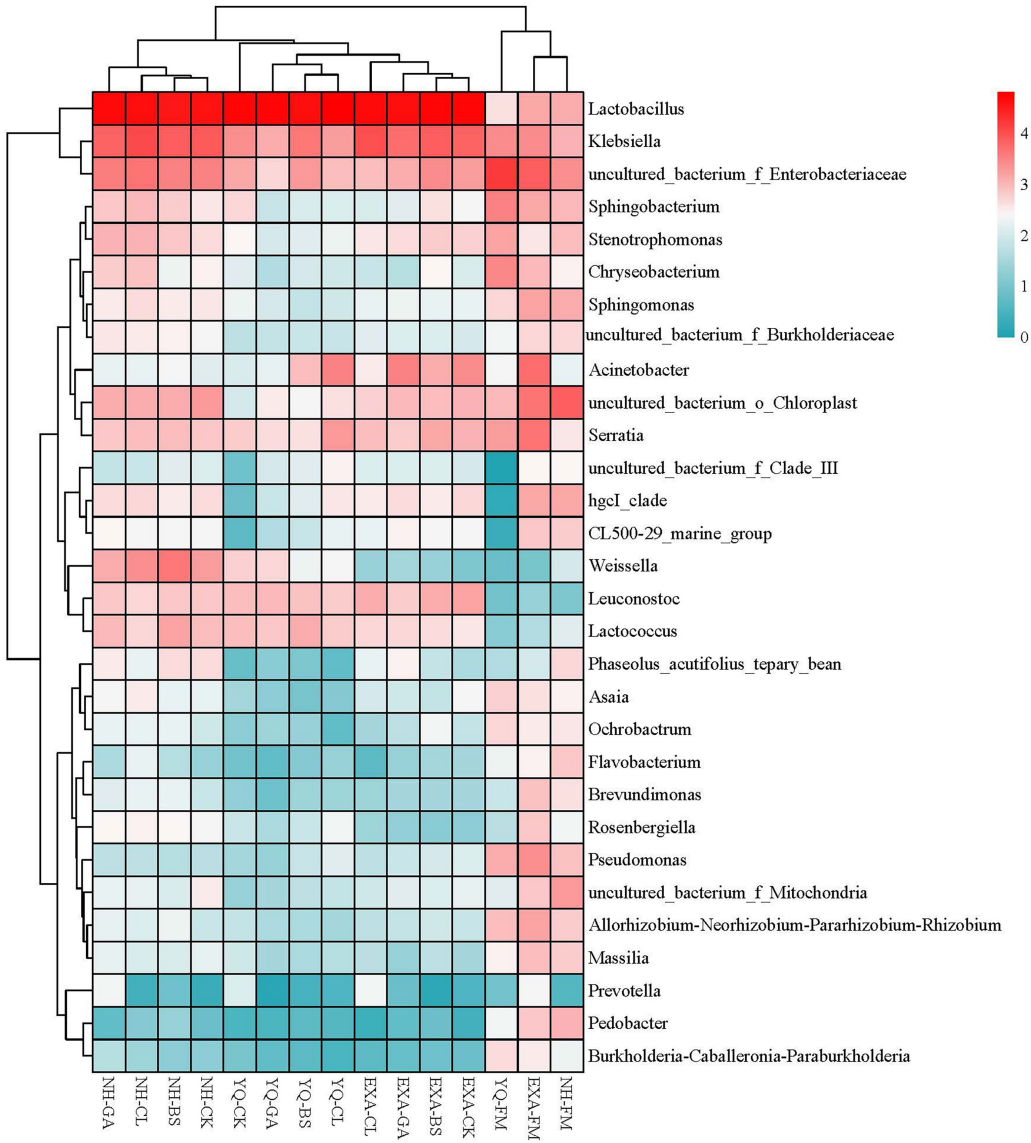
Community heat map of bacterial genera for whole-plant corn silage with different treatments. YQ, corn variety Yuqing 386; EXA, corn variety Enxiai 298; NH, corn variety Nonghe35; FM, fresh raw materials; CK, no additives; BS, 0.02% *Bacillus subtilis* CP7 of FM; GA, 0.2% gallic acid of FM; CL, 0.02% cellulase of FM.

The bacterial community at the genus level was assessed to further show the impact of varieties and additives on the bacterial community in WPC silage (Figures 4, 5). The accumulation columnar map (Figure 4) illustrated the diversity and abundance of bacterial community at various genus levels. *Enterobacteriaceae* (36.62%), *Sphingobacterium* (8.61%), and *Chryseobacterium* (7.16%) were the top three dominant genera attached to YQ-FM; *Enterobacteriaceae* (11.57%), *Serratia* (8.87%), and *Acinetobacter* (7.76%) were the top three dominant genera that attached to EXA-FM; and the top three dominant genera attached to NH-FM were *Chloroplast* (18.49%), *Rosenbergiella* (6.07%), and *Mitochondria* (5.63%). After fermentation, *Lactobacillus* emerged as the dominant genus in each treatment group. In YQ and NH, the relative abundance of *Lactobacillus* was both the highest in the GA group, at 87.56 and 62.18%, respectively, and the lowest both in the BS group, at 73.84 and 49.29%, respectively; in EXA, the relative abundance of *Lactobacillus* was the highest in CK group, and the GA group was the lowest, at 71.01 and 65.68%, respectively. The relative abundance of *Lactobacillus* in the silage of the three corn varieties was ranked YQ > EXA > NH. The relative abundance of *Enterobacteriaceae* decreased significantly in YQ and EXA, remained basically unchanged in NH, and only increased slightly in NH-CL group. In all groups, the relative abundance of *Enterobacteriaceae* was the lowest in YQ-GA group and the highest in NH-CL group, which were 0.92 and 6.62%, respectively. After the silage was completed, *Klebsiella* increased in all groups except the YQ-BS group. The relative abundance of *Sphingobacterium* showed a downward trend after fermentation. Except for the four groups of YQ-CK, NH-BS, NH-GA, and NH-CL, the relative abundance of *Sphingobacterium* in the other groups was at the level of 1%. In addition, corn raw materials were grouped into one category, and WPC silage of three varieties were grouped into one category, respectively. The bacteria of the first three genera of relative abundance (*Lactobacillus*, *Klebsiella*, and *Enterobacteriaceae*) were grouped into one class, and the other different genera were grouped into another class.

## Discussion

### Chemical compositions, lignocellulosic compositions, and fermentation quality of whole-plant corn silage with different treatments

DM content reflects the nutritional value and fermentation quality of whole-plant corn silage, and further determines the economic benefits of silage (Kennington et al., 2005). Khan et al. (2015) reported that corn silage feeding dairy cow with dry matter content in the range 30–35% had a positive effect on improving their milk production. In this experiment, after silage, the content of DM in YQ and NH used different additives were significantly higher than those in the CK group. At the same time, except for the YQ-CK and YQ-BS groups with lower DM content, the DM content of the other treatment groups were within the range 30–35%, this might be a good phenomenon for the dairy cow breading industry. Guo et al. (2013) showed that higher moisture content of silage raw material can lead to *Clostridium*-based fermentation, which produces butyric acid and results in poor silage quality and huge economic losses. The lower DM content of YQ-CK and YQ-BS in this experiment may be because the moisture content of YQ raw material is higher, which leads to the growth of undesired microorganisms and consumes more DM. CP is an important indicator reflecting the nutritional value of silage. WSC is an important microbial fermentation substrate in the silage process. It is generally believed that the content of WSC > 5% DM can meet the needs of microorganisms in the fermentation process, thereby ensuring the fermentation quality (Ni et al., 2017). The WSC content of each type of corn raw materials used in this investigation complied with the aforementioned standards. After silage, in YQ and EXA, the content of CP in BS group was significantly higher than that in CK group, and in YQ, the content of WSC in BS group was significantly lower than that in CK group. Bai et al. (2020) found that the CP content of alfalfa silage increased significantly compared with the CK group after adding *Bacillus subtilis* producing antimicrobial peptide, while the WSC was lower than the CK group, which agreed with the findings of this investigation. This may be the antibacterial peptide produced by the additive can efficiently thwart undesirable bacteria’ physiological activities and promote the activities of LAB, thus consuming a great number of WSC and better protecting CP from degradation, so that the content of CP is relatively increased and the content of WSC is significantly reduced. In this experiment, the content of CP in GA and CL group was significantly higher than that in CK group, and the content of WSC was significantly higher than that in BS group and CK group. Previous studies have shown that gallic acid has anti-fungal, anti-viral, anti-inflammatory and anti-oxidant effects (Choubey et al., 2018; Kahkeshani et al., 2019). This might be the cause of the greater CP and WSC contents in the GA group compared to the CK group. Cellulase can improve the degradation rate of fiber in plant cell wall and produce more WSC as fermentation substrate, this can accelerated the fermentation process by promoting the physiological activities of lactic acid bacteria, protect proteins from degradation by undesired microorganisms, which further improves the content of CP and WSC (Cai et al., 2019). In addition, there were some differences in the contents of DM, CP, EE, ash and WSC of WPC silage of different varieties. The DM contents of EXA and NH were higher, and the CP and WSC contents of YQ and EXA were higher. The research of Cherney et al. showed that there will be differences in the content of chemical components of whole-plant corn silage of different varieties, which is consistent with the results of this study (Cherney et al., 2004). This might be because there were some differences in the growth period of different corn varieties, so the accumulation law of chemical components was also different, resulting in different content of chemical components after silage.

Lignocellulosic components mainly include NDF, ADF, ADL, Cl, HC, and HoC, which are related to the palatability and biodegradability of silage. In this study, the NDF and ADF contents of all varieties of WPC decreased after silage, this was consistent with the research results in whole-plant corn silage with lactic acid bacteria and organic acid by Jiang et al. (2020). Such results indicate that silage process can be used as biochemical pretreatment to promote the degradation of lignocellulose components, so as to realize further utilization (Aichinger et al., 2015; Zhao et al., 2018). The NDF content in YQ, ADF content in EXA and ADL content in NH all showed the same rule that was compared with CK group, the content of this index was significantly reduced after adding gallic acid. In addition, the CEL and HoC contents of GA group were significantly lower than those of CK group in all corn varieties in this experiment. This might be because the use of gallic acid played its acidic role, which could rapidly reduce the pH of silage, thus promoting the acid hydrolysis reaction of NDF, ADF, and ADL. The ADF and ADL contents of CL group were significantly lower than those of CK group in corn variety YQ, CEL, and HoC contents of CL group were significantly lower than those of CK group in all corn varieties. It is explained that the used of cellulase causes an enzymatic reaction in the silage process, which decomposes the cellulose structural carbohydrates in the plant cell wall, resulting in a decrease in the contents of NDF, ADF, HoC, and so on (Desta et al., 2016). BDP is an index to measure the degradability of silage (Agneessens et al., 2015), which is derived from HoC and ADL. In this experiment, in corn varieties EXA and NH, the BDP of each additive group was higher than that of CK group, indicated that several additives used in this experiment were helpful to improve the biodegradability of silage. In addition, in this experiment, ADF, ADL, HC, and CL were different among different varieties WPC silage. Benefield et al. (2006) and Borreani et al. (2018) found that the composition of CP, EE, and other chemical components varied significantly and NDF, ADF, and other lignocellulosic components between different varieties of whole-plant corn silage. This is similar to the results of this experiment. It may be that different corn varieties have different genetic backgrounds, which leads to this difference.

pH, lactic acid, and NH_3_-N / TN are the main indexes to measure the fermentation quality of silage. It is generally believed that high-quality silage has pH <4.2, lactic acid content of 4–6%, NH_3_-N / TN <10% (Wang et al., 1999; Shao et al., 2005; Yuan et al., 2012). In this research, the pH values of all treatment were between 3.8 ~ 4.0, indicated that the WPC is easy to be prepared into high-quality silage. Lara et al. (2016) found that the addition of *Bacillus subtilis* will reduce the LA content in corn silage. In this experiment, the LA content of BS group in YQ and NH was considerably larger than that of CK group and CL group. *Bacillus subtilis* can create LA by pyruvate reduction under anaerobic circumstances, according to Cruz Ramos Hugo et al. (2000), which might account for the elevated LA level in the BS group in our test. It also might be that the addition of antimicrobial peptide-producing BS promoted the proliferation of homofermentative lactic acid bacteria in WPC silage, resulting in more LA (Bai et al., 2020). Among all corn varieties, LA content in GA group was significantly increased than that in CK group, aside from the acidity of GA, it should be owed to its anti-bacterial property benefiting the dominance establishment of lactic acid bacteria and reduced negative nutrient competition (He et al., 2020). In YQ and EXA, compared with CK group, LA content in CL group was significantly increased. Zhao et al. (2021) found that cellulase can increase LA content in silage, in the study of the interaction between cellulase and lactic acid bacteria on the mixed silage of soybean residue and corn straw. Which is consistent with the results of our experiment, it is explained that the structural carbohydrates in the WPC were degraded to a great extent, so that the soluble sugars are released, providing additional fermentation substrates for the fermentation of lactic acid bacteria, thus producing more LA (Stokes, 1992). Furthermore, the presence of PA was not detected at all in this test, and the content of BA was extremely low, which is similar to the study of Wang et al. (2019b) in alfalfa and stylo silage mixed with Moringa oleifera leaves. This indicates that the WPC silage had better quality in this experiment. It is reported that heterofermentative lactic acid bacteria can produce acetic acid and propionic acid during silage (Xu et al., 2021). We speculated that the fact that propionic acid was not detected in this experiment might be due to the absence of heterofermentative lactic acid bacteria activity during silage in this experiment. In principle, protein degradation will not be avoided during silage. This converts total nitrogen (TN) to non-protein nitrogen (NPN) including small peptides, amino acid free nitrogen, and NH_3_-N (He et al., 2020). In addition, ammonia nitrogen is a more accurate indicator of protein hydrolysis, reflecting the deamination of amino acids or peptides (Li et al., 2018). In present study, NH_3_-N / TN of GA group and CL group were significantly lower than that of BS group and CK group among all varieties. Wang et al. (2021) showed that gallic acid can reduce the content of NH_3_-N in whole plant soybean silage, which is consistent with the results of our test. The deamination of peptides or amino acids in silage may be restricted by the GA addition, resulting in less NH_3_-N being produced and greater nutrient protection in WPC silage. FS is calculated by DM and pH, and it is a comprehensive reflection of DM content and pH value (Wang et al., 2017). In this test, FS of each group is greater than 100, which belongs to excellent level. This also shows that WPC is a high-quality raw material for silage production. In all groups, the FS of EXA-GA group was the highest, which also benefited from its higher DM and lower pH. In addition, some fermentation indexes, such as pH, LA, and NH_3_-N / TN, were significantly different among corn varieties. Darby and Lauer (2002) showed that the fermentation quality of whole-plant corn silage would not be different due to different corn varieties. This was different from the results of this experiment, which may be because the corn varieties he selected have the same growth period and similar genetic background, resulting in no difference in the fermentation quality of WPC silage.

### Bacterial community diversity of whole-plant corn silage with different treatments

Silage process is a complex microbial symbiosis system, in which many microorganisms participate. Therefore, its structural composition and diversity affect the fermentation quality and nutrients (Zhou et al., 2016). Bacterial alpha diversity is mainly used to reflect species richness, evenness and sequencing depth (Caporaso et al., 2012), which is represented in diversity (Shannon and Simpson indexes), richness (Chao1 index), and OTUs. In this study, in YQ and EXA, the ACE, and Chao1 index of each additive group were higher than those of CK group, and GA group was the highest. In YQ and NH, Shannon and Simpson index of GA group and CL group were lower than CK group, and both of them were the lowest in GA group. This indicates that GA group has high species richness and low biodiversity. According to records, the greater the abundance of dominant bacteria, the lower the diversity of microbial community, and vice versa (Ogunade et al., 2018). This may be due to the fact that the antibacterial and acidic properties of gallic acid inhibit the activities of non acid tolerant microorganisms, thereby increasing the relative abundance of acid tolerant microorganisms (Xu et al., 2019). In addition, among the three varieties, the ACE, Chao1, Shannon and Simpson index are the lowest in YQ, which indicates that YQ silage has lower species richness and diversity. This may be caused by the difference of microbial composition carried by different varieties of corn silage materials, or the difference of microbial community structure caused by different nutritional composition of different corn silage materials.

The results of principal component analysis can be used to distinguish the bacterial communities of different treatment groups. Fresh WPC materials of three varieties (YQ-FM, EXA-FM, and NH-FM) gathered in the second and third quadrants, and all WPC silage gathered in the first and fourth quadrants. The raw materials of the three corn varieties were clearly separated. Therefore, it can be explained that there are differences in bacterial communities among the three different varieties of WPC raw materials. There were also differences in bacterial communities between WPC silage and corn raw materials. This is due to the anaerobic conditions of silage. Under anaerobic conditions, a large number of microbial life activities attached to fresh corn raw materials were inhibited and gradually replaced by facultative anaerobic and acid resistant lactic acid bacteria (Ni et al., 2017).

### Bacterial relative abundance of whole-plant corn silage with different treatments

Silage is formed by microorganisms community under extremely complex environment, and bacteria play an important role in the whole fermentation process (Li et al., 2019; Yang et al., 2019). In this study, before silage, *Proteobacteria* was the dominant phylum of fresh raw materials of three varieties of corn, followed by *Bacteroidetes* and *cyanobacteria*. After silage, *Firmicutes* evolved into a new dominant phylum in all treatment groups. However, the relative abundance of *Proteobacteria* decreased significantly and became the second dominant phylum, such results are also reflected in the research of Keshri et al. (2018). Previous studies have shown that *Firmicutes* can produce acid and secrete a variety of enzymes in anaerobic environment, and anaerobic and low pH environment help to promote the growth and reproduction of *Firmicutes* (Wang et al., 2018; Ali et al., 2020). In this experiment, after silage, among corn varieties YQ and NH, the relative abundance of *Firmicutes* was the highest in GA group, and the relative abundance of *Proteobacteria* was the lowest in GA group. This may be because the addition of GA rapidly reduces the pH of WPC silage, which provides strong conditions for the growth and reproduction of *Firmicutes*, and promotes the relative abundance of *Firmicutes* in GA group to increase significantly and the relative abundance of *Proteobacteria* to decrease significantly. *Firmicutes* can decompose macromolecules like cellulose and starch (Romero et al., 2017). Therefore, it is inferred that the changes of lignocellulosic composition in this study may also be related to *Firmicutes*. Furthermore, among the three corn varieties, the relative abundance of *Firmicutes* is the highest in YQ, which might be related to the higher WSC content of YQ, which also indicates that corn variety YQ might have higher potential to make silage.

Further, we studied the changes at genus level of bacterial community in WPC silage. Before silage, the dominant bacteria of different varieties of WPC silage raw materials were different. But mainly *Enterbacteriaceae*, *Chloroplast*, *Sphingobacterium*, *Serratia*, *Chryseobacterium*, *Acinetobacter*, *Rosenbergiella*, and *Mitochondria*. However, the relative abundance of *Lactobacillus*, which plays a major role in the silage process, was very low. After silage, the dominant genus in all groups was *Lactobacillus*, followed by *Klebsiella*, *Enterobacteriaceae*, *Acinetobacter*, and *Serratia*, and different groups have different relative abundances. As is known to all, *Lactobacillus* plays a key role in increasing LA and reducing pH, and high-level *Lactobacillus* has also become a symbol of high-quality silage (Mu et al., 2020). In this study, we observed that the addition of GA to corn varieties YQ and NH increased the relative abundance of *Lactobacillus*. This may be because the acidity of GA rapidly lowered the pH of silage or because its antibacterial properties prevented the growth of undesirable microorganisms and created an ideal environment for the *Lactobacillus* to flourish. However, this phenomenon is not shown in the variety EXA, so it is inferred that this may be caused by the amplification of the differences in the genetic background of different corn varieties. *Klebsiella* is a facultative anaerobic bacterium, which is a genus of *Enterobacteriaceae* under *Proteobacteria*. It can produce carbon dioxide and cause a variety of diseases (Podschun and Ullmann, 1998; Wang et al., 2022). In this experiment, *Klebsiella* is the second dominant genus in silage, and previous studies have had similar results (Ogunade et al., 2018). Compared with that before fermentation, the relative abundance of *Klebsiella* only decreased in YQ silage, and the use of BS and GA seemed to promote the growth of *Klebsiella*, which was obviously undesirable. However, it is worth noting that in YQ silage and NH silage, the addition of GA inhibited the growth of *Klebsiella* compared with CK group. Hu et al. (2018) reported that adding mixed organic acid salts to WPC silage can reduce the relative abundance of *Klebsiella*, which might be an effective means to improve this phenomenon. Because *Enterobacteria* can compete with LAB to produce acetic acid during fermentation, resulting in the loss of nutrients, it is undesired in silage (Muck, 2010). Although there was a noticeable decline in relative abundance of *Enterobacteria* in the current research compared to that before silage, the relative abundance of *Enterobacteraceae* was still rather high in silage. Fortunately, in YQ silage and EXA silage, the addition of GA and CL has a certain effect on reducing the relative abundance of *Enterobacteria*. Although *Acinetobacter* requires oxygen, it can rely on acetate to survive in an anaerobic environment (Fuhs and Chen, 1975). Previous studies have reported that the increase of the relative abundance of *Acinetobacter* was associated with the increase of acetic acid content (Ogunade et al., 2017). However, this correlation is not reflected in this study, and the specific reasons need to be further studied. *Serratia* usually produces 2,3-butanediol (McDonald et al., 1991), but no 2,3-butanediol was detected in this study.This is irrelevant, because even if *Serratia* exists, it becomes next to nothing after the fermentation process. Remarkably, the addition of GA seems to have a certain inhibitory effect on the survival of *Serratia*, although this effect is very weak. This was might decided by the antibacterial properties of GA. There was another interesting phenomenon, Parvin et al. (2010) found that *Serratia* can be detected in ryegrass silage, but not in raw materials before silage, which is quite different from the results of this test. It may be attributed to the difference of silage materials or geographical location. After all, forage species, geographical location, maturity, the type of fertilizer used and the competition of epiphytic microbial communities are all factors that affect the spatial structure of microbial communities in silage (McGarvey et al., 2013). It can be seen from the community heat map of bacterial genera (Figure 5) that the grouped clusters were mainly grouped into four categories. The fresh corn raw materials were grouped into one class, and the WPC silage of each variety after silage were grouped into three other classes. This was not only consistent with the previous results of this experiment, but also confirmed that corn varieties had an important impact on WPC silage. Additionally, we noticed that among the three fresh corn raw materials, NH and EXA were classified as one class, YQ was classified as another one class and carried less *Lactobacillus*. However, the relative abundance of *Lactobacillus* in YQ after silage was the highest among the three varieties, suggesting that YQ might had high potential for producing high-quality WPC silage. Overall, we screened out the best corn variety among the three test varieties. Moreover, several additives were helpful to improve the quality of WPC silage, but the most significant effect was GA. This indicated the potential of GA as a new silage additive.

## Conclusion

It is concluded that, after silage, YQ has higher CP, WSC, LA contents and lower NDF, ADF contents; its *Firmicutes* and *Lactobacillus* has the highest relative abundance on the phylum and genus levels, respectively. The three additives increased the contents of DM, CP and LA to a certain extent, reduced the value of NDF, ADF and NH_3_-N/TN, and the value of BDP was relatively higher than that of the CK group; In addition, the used of additives also improved the bacterial community structure, in which the relative abundance of *Firmicutes* on the phylum level and *Lactobacillus* on the genus level were the highest in GA group. The above results showed that YQ quality was the best among the three corn varieties, and GA has the most significant effect on the quality improvement of WPC silage among the three silage additives, which can be recommended to be used in actual operation.

## Data availability statement

The data presented in the study are deposited in the NCBI repository (https://www.ncbi.nlm.nih.gov/), accession number PRJNA882789; The original contributions presented in the study are included in the article/Supplementary material, further inquiries can be directed to the corresponding author.

## Author contributions

ZZhan, YW, LiZ, and YL designed the experiment. ZZhan wrote the original draft. YW contributed to the review of the paper and the acquisition of the funding. ZZhan, BZ, and ZZhai conducted experiments. SW, LuZ, and WJ contributed to the experiment methods and material consult. ZZhan and ZZhai performed data analysis and result visualization. All authors contributed to the article and approved the submitted version.

## Funding

This research was financially supported by the China Agriculture Research System (CARS-38) and the National “13th five-years plan” Key Research and Development Project sub topic of China (2018YFD0502001).

## Conflict of interest

The authors declare that the research was conducted in the absence of any commercial or financial relationships that could be construed as a potential conflict of interest.

## Publisher’s note

All claims expressed in this article are solely those of the authors and do not necessarily represent those of their affiliated organizations, or those of the publisher, the editors and the reviewers. Any product that may be evaluated in this article, or claim that may be made by its manufacturer, is not guaranteed or endorsed by the publisher.

## Supplementary material

The Supplementary material for this article can be found online at: https://www.frontiersin.org/articles/10.3389/fmicb.2022.1028001/full#supplementary-material

## Footnotes

^1^https://qiime2.org/ (Accessed September 9, 2022).
